# Myelin repair in Alzheimer’s disease: a review of biological pathways and potential therapeutics

**DOI:** 10.1186/s40035-022-00321-1

**Published:** 2022-10-26

**Authors:** Lauren Rose Hirschfeld, Shannon L. Risacher, Kwangsik Nho, Andrew J. Saykin

**Affiliations:** 1 Stark Neurosciences Research Institute, Indiana University School of Medicine, Indianapolis, IN USA; 2 Indiana Alzheimer’s Disease Research Center, Indiana University School of Medicine, Indianapolis, IN USA; 3 Department of Radiology and Imaging Sciences, Indiana University School of Medicine, Indianapolis, IN USA; 4 School of Informatics and Computing, Indiana University-Purdue University Indianapolis, Indianapolis, IN USA

**Keywords:** Myelin, Alzheimer’s disease, Myelin repair, Oligodendrocyte, Remyelination

## Abstract

This literature review investigates the significant overlap between myelin-repair signaling pathways and pathways known to contribute to hallmark pathologies of Alzheimer’s disease (AD). We discuss previously investigated therapeutic targets of amyloid, tau, and ApoE, as well as other potential therapeutic targets that have been empirically shown to contribute to both remyelination and progression of AD. Current evidence shows that there are multiple AD-relevant pathways which overlap significantly with remyelination and myelin repair through the encouragement of oligodendrocyte proliferation, maturation, and myelin production. There is a present need for a single, cohesive model of myelin homeostasis in AD. While determining a causative pathway is beyond the scope of this review, it may be possible to investigate the pathological overlap of myelin repair and AD through therapeutic approaches.

## Background

Alzheimer’s disease (AD) is the leading cause of dementia and currently has few avenues of effective treatment. AD has been well-characterized as a disease involving primary pathologies of intracellular neurofibrillary tau tangles and extracellular amyloid beta (Aβ) plaques. Pharmaceuticals have been developed to target these hallmark pathologies, but even therapeutics successful at reducing Aβ plaque load have been unable to sufficiently halt disease progression. Recently, it was proposed that the only FDA-approved amyloid-targeting drug, Aducanumab, may be more efficacious when combined with drugs that target oligodendrocytes and encourage remyelination [1].

Myelin is the lipid-based sheathing which surrounds axons to protect and promote nerve conduction. Demyelination has been observed in vivo in patients with mild cognitive impairment and dementia using myelin water fraction (MWF), a sophisticated neuroimaging method that is preferentially sensitive to myelin, indicating its potential utility as a clinical biomarker for dementia [2, 3]. Additionally, myelination in the elderly without dementia, measured through MWF, has been associated with episodic and semantic memory capacity [4] and the AD risk allele apolipoprotein E (*APOE*) ε4 [5]. In cognitively normal older adults at risk for AD, evidence supports the association of MWF with cerebrospinal fluid biomarkers of AD such as phosphorylated tau 181, total tau, and Aβ [6]. Thus, it is possible that promyelinating strategies may potentially ameliorate hallmark AD pathology and cognitive decline.

**Fig. 1:**
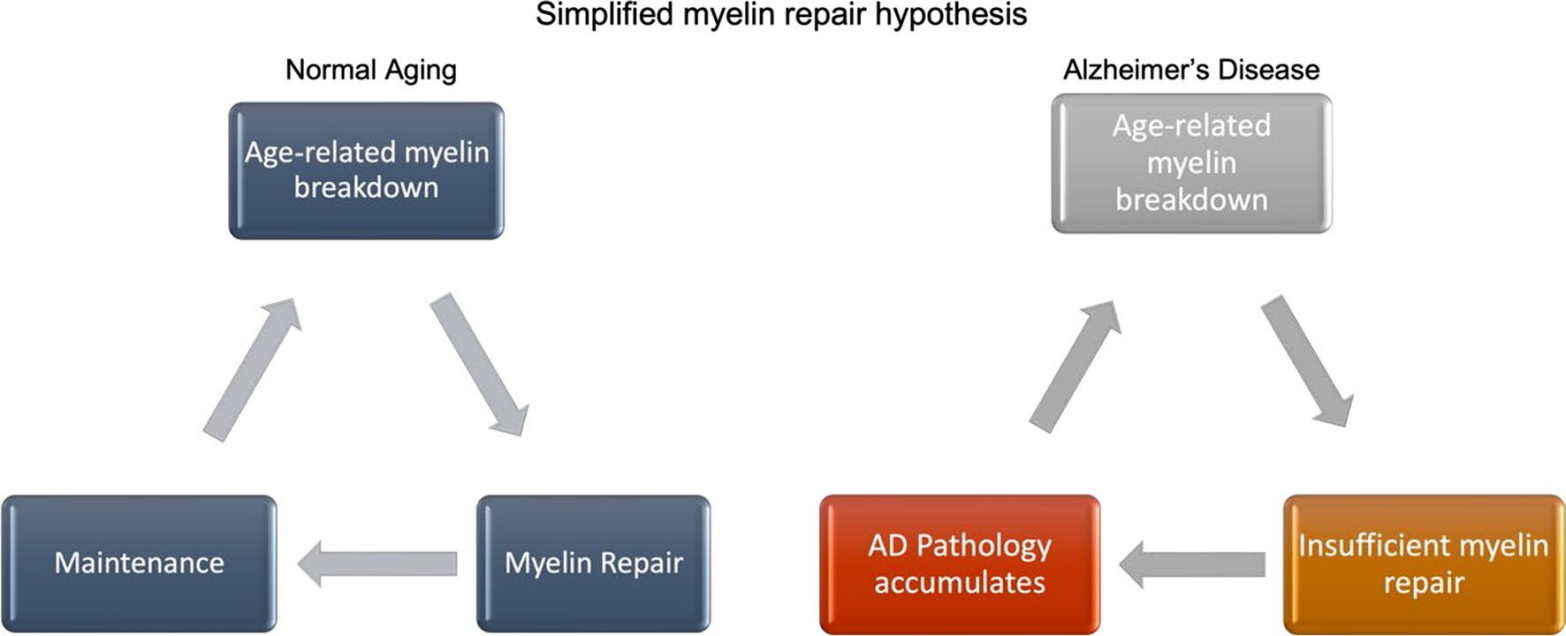
Dysfunctional myelin repair mechanisms in individuals with AD, which may interrupt the normal myelin repair and maintenance cycle and lead to downstream amyloid-beta deposition and tau pathology as previously proposed by Bartzokis et al. [4]

In considering potential therapeutic targets, early research by George Bartzokis and colleagues on myelin in AD and the association of Aβ with late-myelination regions is especially relevant [7–9]. Bartzokis proposed the theory that Aβ and tau are secondary, rather than primary, pathologies and may result from the brain attempting to maintain myelin homeostasis through a cycle of damage, repair, and maintenance [9] (Fig. 1).

In his hypothesis, Bartzokis theorizes that the complexity of human evolution and the related development of sophisticated myelination put humans at a unique vulnerability to neurological diseases stemming from the breakdown of and subsequent effort to maintain myelination [9]. Many pathways involved in myelin damage, maintenance, and repair overlap with genetic correlates of AD. However, demyelination and injury alone are not sufficient to cause AD pathology [9–12]. Typical myelin maintenance patterns follow a well-characterized, normal, age-related, quadratic trajectory [7, 13–15], which suggests that age-related pathology may not be due to mechanisms of myelin damage or maintenance but rather occur in the attempts to repair myelin. Although an increase in oligodendrocyte progenitor cell (OPC) proliferative rate suggestive of endogenous repair has been observed in an AD mouse model, this finding was not observed in post-mortem AD brain tissues, further suggesting insufficient repair mechanisms unique to clinical AD [16].

In this comprehensive review, we focus on the overlaps of myelin repair pathways empirically demonstrated to induce remyelination and AD-relevant pathways, to identify targetable mechanisms for potentially preventing AD progression. Other therapeutics that act on the intersection of myelin repair and AD pathology that should be investigated further are also reviewed.

## Search criteria

The following keywords and terms were used to search for literature within the scope of this review: (myelin repair) AND (tau); (myelin repair) AND (amyloid); (myelin repair) AND (apoe4); (myelin repair) AND (apoe); ((myelin repair) AND (alzheimer's)) AND (gene); (myelin repair) AND (alzheimer's); (remyelination) AND (alzheimer's); ((remyelination) AND (alzheimer's)) AND (gene); (remyelination) AND (apoe); (remyelination) AND (apoe4); (remyelination) AND (amyloid); (remyelination) AND (tau); (myelin repair) AND (iron); (remyelination) AND (iron); ((myelin repair) AND (alzheimer's)) AND (iron); ((remyelination) AND (alzheimer's)) AND (iron).

The initial search yielded 318 articles after removal of duplicate retrievals. Exclusion criteria included: publication before 1992, review article or meta-analysis, organisms other than humans or murine, studies that did not have empirical evidence of affecting remyelination or myelin repair, studies that only investigated protection, injury, or early development of myelin, and studies not related to AD. After removal based on these criteria, 68 studies remained and are covered in this review. If studies covered myelin repair with indirect relationships to AD pathology, citations and PubMed were further searched for relevance to AD before exclusion was determined. Reviews were cited if the gene or pathway target has been thoroughly researched beyond the scope of this review. These parameters yielded another 50 results, bringing the total references cited to 118.

## Examining overlaps between myelin repair and AD signaling pathways

### Amyloid precursor protein (APP) and Aβ

Myelin pathology has been demonstrated to co-localize to Aβ plaque deposition in a commonly used AD mouse model with 5 familial AD mutations (5× FAD). The model, which expresses AD-associated mutations in *APP* and presenilin 1 (*PSEN1*) genes, is reportedly absent of confounding tau pathology [17], supporting the association of Aβ and myelin pathology. Additionally, soluble Aβ oligomers have been shown to: (a) specifically inhibit the survival of mature oligodendrocytes (OLs), cells that mainly function to assemble myelin sheathing, and (b) prevent myelin sheath formation [18]. In contrast, soluble Aβ oligomers can also induce myelin basic protein (MBP) expression, a vital protein in myelination, as well as promoting OL differentiation and maturation [19]. When Aβ toxicity is reduced, myelin integrity is rescued, but regeneration is not affected [20, 21]. Low-sulfated modified heparin mimetics have been shown to bind to Aβ and specifically prevent it from inhibiting OPC differentiation into mature OLs, leading to rescued remyelination [22].

Upstream of Aβ plaque deposition is APP, which is necessary for myelin repair, as knocking out APP leads to very delayed or no remyelination [23]. In a transgenic mouse model that overexpresses amyloid pathology, early disease stages of Aβ plaque deposition are associated with increased OPCs and their subsequent differentiation into mature OLs [16]. On the other hand, too much APP may also impair remyelination, as the same study also found decreased OLs in analogous human AD post-mortem tissues. A possible mechanism of myelin repair may be tied to Arginase 1 (Arg1) expression*,* as bulk RNA transcriptome analysis and cell type-profiling of APP mice demonstrated a significant association between insufficient Arg1 expression in myeloid cells, including OLs and other glial and phagocytic cells, and subsequent neurodegeneration and Aβ deposition [24]. Counterintuitively, Arg1 deficiency promotes OLs; more expectedly, it upregulates pro-inflammatory markers. Arg1 is also known to be significantly reduced in demyelination [25], further implicating Arg1 deficiency in overall myelin pathology.

APP is processed through several downstream pathways that may mechanistically explain the protein’s impact on myelination. First, APP can be processed by a family of α-secretases, known as a disintegrin and metalloproteinase (ADAM, such as ADAM10 and ADAM17), to form the non-neurotoxic soluble APP alpha (sAPPα) [26]. Upregulated sAPPα appears to be not just repair-oriented, but also protective in a demyelinating context. The administration of a sAPPα promoter, etazolate, in a mouse model of demyelination subsequently restored damaged myelin, upregulated MBP and mature OLs, and protected from further demyelination [27]. sAPPα may be targeted with the FDA-approved acetylcholinesterase inhibitor (AChEI) rivastigmine, which has been shown to encourage α-secretase processing activity in both 3× TG mice and human post-mortem tissues [28]. However, there is evidence that rivastigmine does not directly affect oligodendrogenesis [29].

APP can also be cleaved by β-secretase, known as BACE-1, which cleaves APP into Aβ [30]. The β-secretase also cleaves neuregulin 1 (NRG1) proteins, which are necessary for initiating remyelination [31, 32]. Aged APP/PSEN1 transgenic mice with vascular pathology, as well as their non-APP aged, stroked counterparts, demonstrated chronic upregulation of BACE1/NRG1 expression, as well as increased amyloid pathology [33]. Selective deletion of BACE1 leads to subsequent NRG1 loss in peripheral injury [34, 35], suggesting the effects of BACE1 signaling on remyelination may be mediated through the neuregulin family. While NRG1 can also be cleaved by ADAMs, specific cleavage by BACE1 seems both necessary and sufficient for NRG1 to signal myelin production [36]. However, bypassing this pathway is possible, as promoting downstream protein kinase B (Akt) expression in OLs rescues  the NRG1-associated production of myelin in a BACE1-deficient model [37]. On the other hand, remyelination does not occur even in the presence of BACE1 in an APP-knock out (KO) model [23].

γ-Secretase also cleaves Aβ [38], and inhibition of γ-secretase can likewise decrease Aβ levels [39]. γ-Secretase inhibition also promotes remyelination, encourages stem cell maturation [40], and is associated with significantly quicker disease recovery and milder pathology in mice with experimental autoimmune encephalomyelitis (EAE), a demyelinating animal model similar to multiple sclerosis (MS) [41]. Inhibition of γ-secretase specifically inhibits the Notch1 signaling pathway, which in turn creates a pro-myelinating environment [41].

Taken together, Aβ and upstream APP may have both pro- and anti-remyelinating properties that are pathway-dependent. sAPPα, BACE1, and γ-secretase are known to affect the deposition of toxic Aβ oligomers in AD, and evidence suggests that these pathways may play a larger role than previously thought in myelin regulation in AD. Additionally, vascular pathology has been shown to contribute to general myelin pathology outside of AD [33, 42], though amyloid pathology appears to exacerbate the injurious effects of vascular injury on myelin [33]. Finally, the myelin repair pathways may be more effective initially in younger APP models [33] regardless of vascular contribution [16]. Further studies are needed to determine to what degree the amyloid and myelin changes are linked both spatially and temporally, as current literature suggests that AD-related demyelination occurs in a heterogenous manner in relation to amyloid pathology [16, 43–45].

In summary, APP and remyelination have significant overlaps in pathway biology. Solely targeting Aβ has been unsuccessful to date in stopping disease progression. Upregulating pro-myelinating pathways and downregulating Aβ concurrently may result in amelioration of pathology beyond what current Aβ clearance drug trials have demonstrated. Future studies testing this hypothesis are warranted.

### ApoE and lipid metabolism

ApoE is a potential key player overlapping several neurodegenerative conditions in which myelin is affected (for review see [46]). *APOE* genotype*,* especially the ε4 allele, has been associated with multiple sclerosis (MS, a myelin-centric disorder) [47, 48], as well as lower apparent diffusion coefficient and fractional anisotropy values by diffusion tensor imaging in normal populations, indicating impaired white matter [49]. *APOE* ε4 carriers have shown developmental differences in white matter and cognition compared to non-carriers [15, 50]. The *APOE* ε2 allele is also associated with impaired remyelination in MS [51], though this finding has not been replicated. In fact, the ε2 allele has been shown to be protective against AD [52, 53] and is associated with higher myelin content compared to noncarriers, as observed via MWF [15]. A worse disease trajectory in *APOE*-deficient EAE mice compared with EAE controls, along with inhibited remyelination with concomitant immune activity, has been observed [54], potentially because the microglia and macrophages become overloaded by the large amount of cholesterol resulting from injured myelin and subsequent breakdown, which leads to their inability to keep up with phagocytic activities to drive downstream remyelination [55]. ATP-binding cassette transporter A1 (ABCA1) is a key player in cholesterol transport and metabolism (for reviews see [56–58]). ABCA1 is essential for astrocytic and glial synthesis of ApoE [59], and is also involved in transporting cholesterol from cells to high-density lipoproteins (HDLs), of which ApoE is a component [59, 60]. *ABCA1*-deficient mice exhibit significant and sustained reductions of OLs and myelin density, and reduced oligodendrogenesis post-stroke; ApoE2 and HDL3 expression directly rescues neurological deficits, promotes OPC differentiation, and significantly attenuates reductions in myelin, OLs, and oligodendrogenesis [61]. ApoE also binds to microglia-expressed triggering receptor expressed on myeloid cells 2 (TREM2) (for review see [62]). TREM2, a rare variant strongly associated with AD [63], is present in early cell proliferation and has been shown to co-localize with OPCs and OLs in *APP/PSEN1* mice [64]. TREM2 is related to myelin repair as it is necessary for the formation of lipid droplets through cholesterol esterification [65]. Additionally, TREM2 plays an essential role in mediating the phagocytosis of myelin and other cellular debris [66, 67]. Single-cell RNA sequencing in mice has identified a TREM2-dependent white matter-associated microglial phenotype (WAM), which has been observed to clump with myelin debris and is involved in debris degradation, hypoxia-inducible factor signaling, and lysosomal and cholesterol pathways [68]. WAM also overlap in genetic signature with disease-associated microglia (DAM), an abnormal microglial type previously observed in transgenic AD mice [69]. While WAM are TREM2-dependent, they are typically *APOE*-independent in wild-type mouse models of aging. However, in mouse models of AD, ApoE is necessary for WAM development in addition to TREM2 [68]. ApoE is also related to the regulation of the enzyme lipoprotein lipase (LPL). LPL, which mediates the reparative phenotype of microglia, is specifically involved in the uptake and phagocytosis of myelin-related lipids, and it is associated with the initiation of improved demyelination-related clinical symptoms in EAE-induced mice at the temporal junction where demyelination ends and remyelination begins [25]. LPL deficiency has been thoroughly investigated as a possible contributing factor in the development of AD [25, 70–72]. LPL administration results in elevated cellular *Arg1* levels [25], which has been previously implicated in myelin repair [24]. Lipid uptake may also be mediated by colony-stimulating factor 1 receptor, which, when inhibited, reduces microglia but potentially enhances the phagocytic capacity of remaining microglia, thus enabling remyelination [73]. Evidence also shows that remyelination may be encouraged through the upregulation of specific lipid receptors such as liver X receptors (LXR), oxysterol-activated nuclear receptors that maintain cholesterol homeostasis. These receptors are present in oligodendrocytes and have been demonstrated to enhance lipid transfer from other cells to OLs when LXR is activated [55, 74]. LXR agonists have been shown to improve remyelination, reduce inflammation, and reduce overall cholesterol overload that occurs in demyelination, through inducing target genes including *ABCA1, APOE,* and others [55, 74]. The nuclear receptor retinoid X receptor (RXR) signaling can similarly upregulate the expression of *ABCA1* and *APOE* to directly increase OPC and OL maturation and improve AD-related cognitive functioning [75].

ApoE mimetics have been able to rescue myelin repair while also suppressing macrophage activity in the peripheral nervous system [76]. Inhibition of low-density lipoprotein receptor 1, an essential receptor for myelin phagocytosis [77], blocks mimetic effects, indicating a potential role for this receptor. LXR and RXR pathways can also be upregulated by synthetic agonists. RXR is a clinically applicable target, and the FDA-approved RXR agonist, bexarotene, is associated with remyelination in triple transgenic (3× TG) mouse models of AD [75] and remyelination-associated cognitive recovery in stroked mice [75, 78]. Additionally, TREM2 has the potential to enter the brain from peripheral sites and directly modulate OPC and OL activity [64].

Taken together, modulation of ApoE-related signaling pathways seems to both improve cognition and encourage remyelination in the context of AD as well as other models of demyelination and vascular injury.

### Tau and neurofilament proteins

**Fig. 2:**
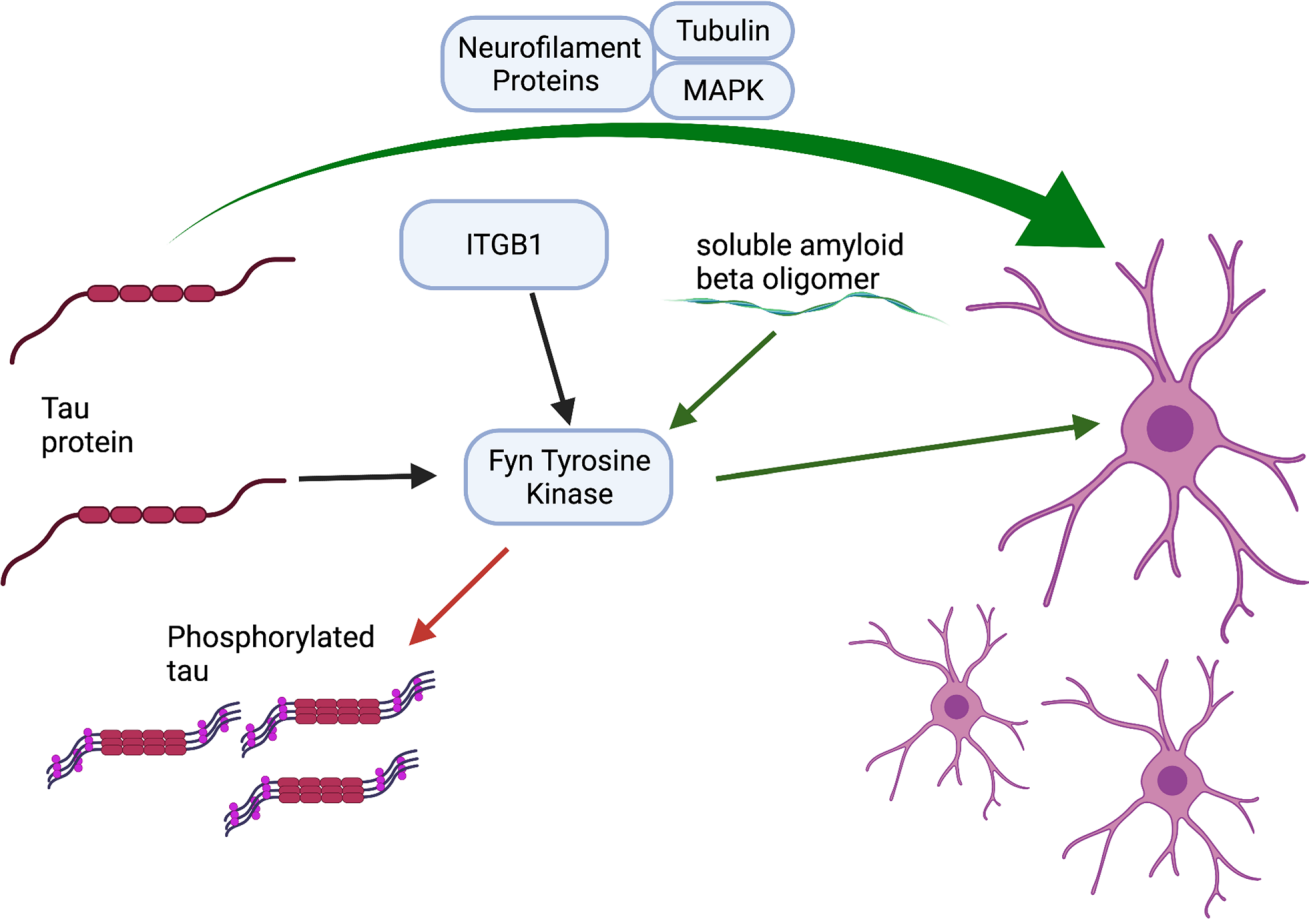
Simplified rendering of tau-associated pathways that may influence OL differentiation, including the binding of tau and Fyn to the OL cytoskeleton, which may simultaneously encourage hyperphosphorylation of tau, an upstream activator of Fyn, ITGB1, and other axonal and neurofilament proteins that may interact with tau to also promote OL differentiation beyond Fyn

Selective myelin injury occurs early in tauopathy models followed closely by cognitive deficits, and in turn, remyelination has been shown to ameliorate cognitive decline [79]. Tau, which becomes hyperphosphorylated and aggregates as neurofibrillary tangles in AD, has been shown to modulate OPC differentiation along with other axonal cytoskeleton proteins like tubulin and microtubule-associated proteins [80, 81]. In addition, tau has been shown to bind to the cytoskeleton of OLs via the truncating tyrosine kinase Fyn [82] (Fig. 2), which is also involved in the phosphorylation of tau [83]. When phosphorylated tau (p-tau) is specifically reduced without affecting the total tau levels, myelin repair is increased and functional outcomes are improved [84]. Tau is also associated with axonal neurofilament proteins (NFPs). Specific fractions of NFP, such as NFP2 and NFP5, have been associated with OL lineage and development in vitro [81]. The ratio of NFP to tubulin may specifically affect OL lineage. NFP2, which is associated specifically with OPC proliferation, contains a higher tubulin concentration. NFP5, which affects OL maturation and promotes differentiation, contains less tubulin [81]. Interestingly, soluble Aβ oligomers can promote OL differentiation/maturation and induce MBP expression through the oligodendrocytic Fyn/Ca2/CAMKII signaling cascade and its upstream activator ITGB1, suggesting that Fyn may serve as a target for simultaneously modulating oligodendrocytic machinery and tau hyperphosphorylation [19] (Fig. 2).

## Exploring other targets implicated in both myelin repair and AD

### Phosphoinositide 3-kinase (PI3k)/Akt/mammalian target of rapamycin (mTOR) signaling

The PI3k signaling cascade and its upstream and downstream components can promote remyelination and overlap with pathways that contribute to AD pathology. Akt expression, downstream of PI3k, was previously discussed in the context of BACE1 cleavage, where Akt expression may serve as a “bypass” to allow BACE1 inhibitors to suppress amyloid without affecting remyelination [37]. Activating the PI3k pathway directly or indirectly has been shown to promote remyelination [85–87]. The traditional Chinese herbal compound Shen-zhi-ling (SZL) oral liquid has been shown to increase PI3k and downstream Akt mRNA expression, as well as significantly increasing mTOR-positive cells and myelin-related proteins in APP mice [88]. Similar effects have also been seen with donepezil, an AChEI that has been approved by the FDA for treatment of AD and has also been investigated for myelin-promoting properties. Donepezil promotes differentiation of OPCs to OLs, encourages the formation of myelin sheathing, and upregulates myelin-specific proteins [29, 89]. Notably, rivastigmine did not have a significant effect on OPCs and myelination when compared to donepezil. While the exact remyelinating mechanism of donepezil is presently unclear, the PI3k/Akt/mTOR pathway modulation is thought to be a contributor [89]. In contrast, low doses of a PI3k antagonist can upregulate OPCs and encourage OL maturation, though it has yet to be determined whether this is a PI3k-specific effect or a result of off-target Wnt and RAF-MAPK signaling [90]. In summary, PI3k/Akt modulation may be useful for promoting remyelination specifically in the context of AD. Further investigations of PI3k and especially Akt in the dual contexts of myelination and AD may offer insight into relevant biological pathways that contribute to AD.

### Histamine receptors

Clemastine, an FDA-approved H1 antihistamine with anti-inflammatory and anti-muscarinic effects, has been shown to reduce Aβ deposition and ameliorate cognitive deficits while simultaneously enhancing OPC differentiation and maturation, as well as myelin integrity, in mouse models of AD [91, 92]. Clemastine can additionally upregulate mTOR to inhibit OPC senescence. OPC senescence has been associated with Aβ in mouse models of AD and in post-mortem brain tissues of AD patients, implicating a possible therapeutic role for senolytic and senescence-inhibiting therapeutics in inducing myelin repair in the context of AD [93].

Histamine 3 receptor (H3R) antagonists and inverse agonists may also be a potential target, as H3R antagonism has been shown to reduce Aβ load, possibly through cAMP response element-binding protein (CREB)-mediated autophagy [94, 95], and improve memory deficits [96]. H3R inhibition acts on the cAMP/CREB/HDAC-1/HES-5 signaling cascade and has been shown to improve remyelination by promoting OL differentiation and maturation by reducing cAMP. Hes Family BHLH Transcription Factor 5 (HES-5) inhibition alone may ameliorate OPC differentiation [97]. HES-5 is also a downstream product of the Notch signaling pathway, which is cleaved by both the ADAM metalloprotease family and γ-secretase (for review see [98]). Abnormal Notch signaling and related *NOTCH* gene mutations are associated with tau and Aβ as well as vascular components of AD (for review see [99]). Notch is also involved in myelination, as inhibition of the Notch signaling pathway in OLs results in quicker recovery and milder clinical manifestations in a demyelinating context, as well as upregulated remyelination [41].

Clemastine has been assessed in a trial in MS patients for its remyelinating properties [100], and it potentially proves useful for AD as well [1]. Additionally, the H3R selective antagonist/inverse agonist, Pitolisant, is FDA-approved to treat narcolepsy with cataplexy, demonstrating feasibility of targeting H3R. In conclusion, selectively targeting histamine, and in turn mediating Notch signaling, may offer a clinically relevant pathway for both myelination and AD.

### Acid sphingomyelinase (ASM)

KARI201, an ASM inhibitor, has been recently developed and may have direct effects on both AD pathology and OL-lineage effects. KARI201 normalizes ASM activity without affecting protein level of ASM or mRNA transcript level of *SMPD1*, the gene coding for ASM. However, this drug is associated with significantly reduced Aβ levels and improved autophagic and phagocytic microglial activity [101]. ASM inhibition also encourages OL maturation and/or survival [102]. KARI201 is also found to have a dual action as a ghrelin receptor agonist; agonism of this receptor is known to promote hippocampal synaptic density, plasticity, and neurogenesis in the context of AD [101].

Other approved or experimental drugs also target the ASM pathway. Amitriptyline is an FDA-approved tricyclic antidepressant that is a potent ASM inhibitor [103], which could be explored further for effects on OL pathways. Though still experimental, mesenchymal stem cell exosomes have been found to directly stimulate OPC proliferation, maturation, and remyelination while also encouraging reparative microglial phenotypes [104]. Moreover, sphingomyelin-driven neuronal exosomes can specifically bind and sequester Aβ [105], which can then be phagocytosed [106], indicating a larger role for sphingomyelin metabolism as a therapeutic target.

### Growth arrest-specific protein 6 (GAS6)

GAS6 has been associated with clinical AD [107] and clearance of Aβ [108]. Additionally, direct delivery of recombinant human GAS6 (rhGAS6) protein to the corpus callosum in demyelinated mice demonstrated a beneficial effect on the clearance and reduction of myelin and lipid debris and encourages accelerated maturation of OPCs [109]. Microglia are not directly affected by rhGAS6, but based on the amelioration of debris clearance in previous studies, it appears that GAS6 may promote microglia to target Aβ, as well as encouraging existing microglia to clear Aβ instead of myelin and/or to restore reparative phagocytic properties in disease contexts [110].

### Klotho

Enhanced expression of the anti-aging gene *KLOTHO* [111] reduces AD-related cognitive deficits [112]. The *KLOTHO-*VS allele heterozygosity has been shown to reduce amyloid and tau pathology [113] in *APOE* ε4 carriers [114]. In addition to its AD-specific effects, Klotho enhances remyelination [115] and promotes OL maturation [116, 117]. Taken together, Klotho appears to specifically encourage myelin repair pathways while also ameliorating amyloid and tau pathology. The recent advance of a CRISPR-Cas9 model for studying *KLOTHO* activation will allow for further research into this gene and its effects [118].

### Phosphodiesterase-5 (PDE5) inhibition

PDE5 inhibition has also been investigated in AD animal models as a potential treatment. Specifically, a rat model of AD treated with a PDE5 inhibitor showed increases in vascular endothelial growth factor A and cyclic GMP, decreases in vascular cell adhesion molecule 1 and tumor necrosis factor alpha, and increased memory performance compared to the non-treated group [119]. Additionally, PDE5 inhibition has been shown to encourage remyelination in demyelinating mouse models while also exhibiting immune-modulating effects [120–122]. These studies indicate that sildenafil, an FDA-approved PDE5 inhibitor, commonly known as Viagra, may have an ameliorating effect on AD pathology along with positive effects on myelination. However, a study of sildenafil treatment of OPCs showed evidence that PDE5 inhibition may also negatively impact myelin gene transcription and impair oligodendrocyte proliferation [123]. Further clinical studies are warranted due to the differing conclusions between in vitro and in vivo studies, which suggest that PDE5 inhibition alone may not be sufficient to induce remyelination, and immune modulation may be necessary with this treatment.

### Nogo-A signaling pathway

Dl-3-butylphtalide (dl-NBP) can ameliorate neuropathology related to AD [124] and may ameliorate myelin injury in vascular models. Specifically, dl-NBP has been shown to promote OPC proliferation through the neurite outgrowth inhibitor (Nogo-A) and brain-derived neurotrophic factor signaling pathways [125]. Nogo-A-deficient mice demonstrate delayed OL maturation and myelination [126]. Leucine-rich repeat and Ig domain containing 1 (LINGO-1), downstream of Nogo-A, directly interacts with and encourages degradation of APP [127], while also negatively impacting myelination and OL differentiation [128]. In contrast, anti-LINGO-1 therapy has been shown to improve spatial learning and, at least partially, to restore MBP levels [12], and genetic deletion of LINGO-1 in animal models also results in enhanced remyelination [129–131]. Relevant to AD, anti-LINGO-1 therapy in a 5 × FAD mouse model ameliorated memory impairment before abnormal Aβ deposition was apparent [132]. In an APP/PS1 mouse model, anti-Lingo-1 therapy decreased the existing Aβ deposition and restored OL function [133]. Single-cell transcriptome analysis of post-mortem human AD brain tissue has further implicated LINGO-1 as a regulator of myelin integrity across not just OPCs and OLs, but also most neuronal and glial cell types, which may reflect a central response attempting to restore myelin homeostasis [134]. Additionally, single-nucleus transcriptome analysis has indicated upregulated LINGO-1 in astrocytes of AD prefrontal cortical samples [128]. Taken together, it appears that modulating the Nogo-A signaling pathway and the downstream LINGO-1 activity may prevent AD pathology and simultaneously encourage OPC proliferation, possibly enhancing remyelination.

### Protein-tyrosine phosphatase receptor type Z (PTPRZ) signaling

PTPRZ acts on the same target in OLs as Fyn; however, it functions conversely. Inhibition of PTPRZ has been shown to upregulate OPC differentiation, as PTPRZ potentially prevents OPC maturation [135]. In addition to competing with Fyn, as discussed previously for its effects on tau, PTPRZ expression is also mediated through metalloproteinase and γ-secretase pathways [136]. PTPRZ, which overlaps with both tau and amyloid processing pathways, along with its direct effects on OPC differentiation, merits further investigations of its effects in relation to AD.

### Iron homeostasis

Iron homeostasis is essential for glial function and myelination, and multiple recent literature reviews implicate dysfunctional iron homeostasis in the pathogenesis of AD [137–139]. Additionally, clinical neuroimaging studies have demonstrated the association between iron accumulation and myelination in normal aging [140, 141] as well as the association between tau accumulation and iron in AD patients [142]. Cerebrospinal fluid levels of iron transport proteins are also associated with cognitive decline in AD [137].

The literature is divided on the benefits of inhibiting or activating iron storage and transport proteins. For example, the iron storage protein ferritin is known to encourage remyelination and OL function through microglial H-ferritin release [143, 144], as OLs themselves express an H-ferritin receptor known as T cell immunoglobulin and mucin domain-containing protein-2 (TIM-2) [145]. H-ferritin iron storage impairment in OPCs and deletion of astrocytic H-ferritin are also associated with a delay in myelin repair [146, 147]. However, despite observations of OL proliferation in inflammatory environments, toxicity of excess ferritin to OL lineage has also been observed [143]. Inflammatory milieu such as the presence of activated microglia and associated cytokine production may also play a role in inducing ferritin toxicity to OLs [144]. Additionally, excess iron in animal models can increase APP, inhibit α-secretase cleavage, and increase β-secretase cleavage [137]; however, APP, in turn, can stabilize the iron export protein ferroportin (Fpn) [137, 148], which is involved in OL maturation and myelination [149]. Another iron import protein, Divalent metal transporter 1, is involved in APP processing and co-localizes with Aβ plaques [150], and its deletion is associated with reductions in OPC maturation and myelination [147]. Transferrin, another iron importer, binds with tau [151] and has been associated specifically with p-tau in AD patients [152]. It also enhances microglial phagocytic capacity, improves lipid and myelin debris uptake, and encourages OL maturation [153, 154].

While the overall upregulation of proteins related to iron homeostasis is evidently associated with AD pathology, the overall downregulation may negatively impact myelination. Modulation of iron and associated transport proteins could be achieved through iron chelators modified to cross the blood–brain barrier and has previously been clinically trialed in several neurodegenerative diseases (for review, see [155]), including AD [156, 157]. Studies examining combined therapeutics that act through iron accumulation and transport to encourage microglial and oligodendrocyte function while also mediating APP cleavage and tau phosphorylation are still needed.

## Summary

This comprehensive literature review highlights the overlaps of signaling pathways involved in myelin repair and AD. Evidence from this review also suggests that dysfunctional myelin repair may occur early in the disease spectrum, and thus, it may be more beneficial to target myelin repair in early disease stages. Further investigation is needed to confirm the spatial and temporal relationship of insufficient myelin repair with AD pathology. The accumulation of amyloid and tau pathology that occur after myelin pathology [79, 132] suggests that early targeting of remyelination could be a potential therapeutic choice.

**Fig. 3:**
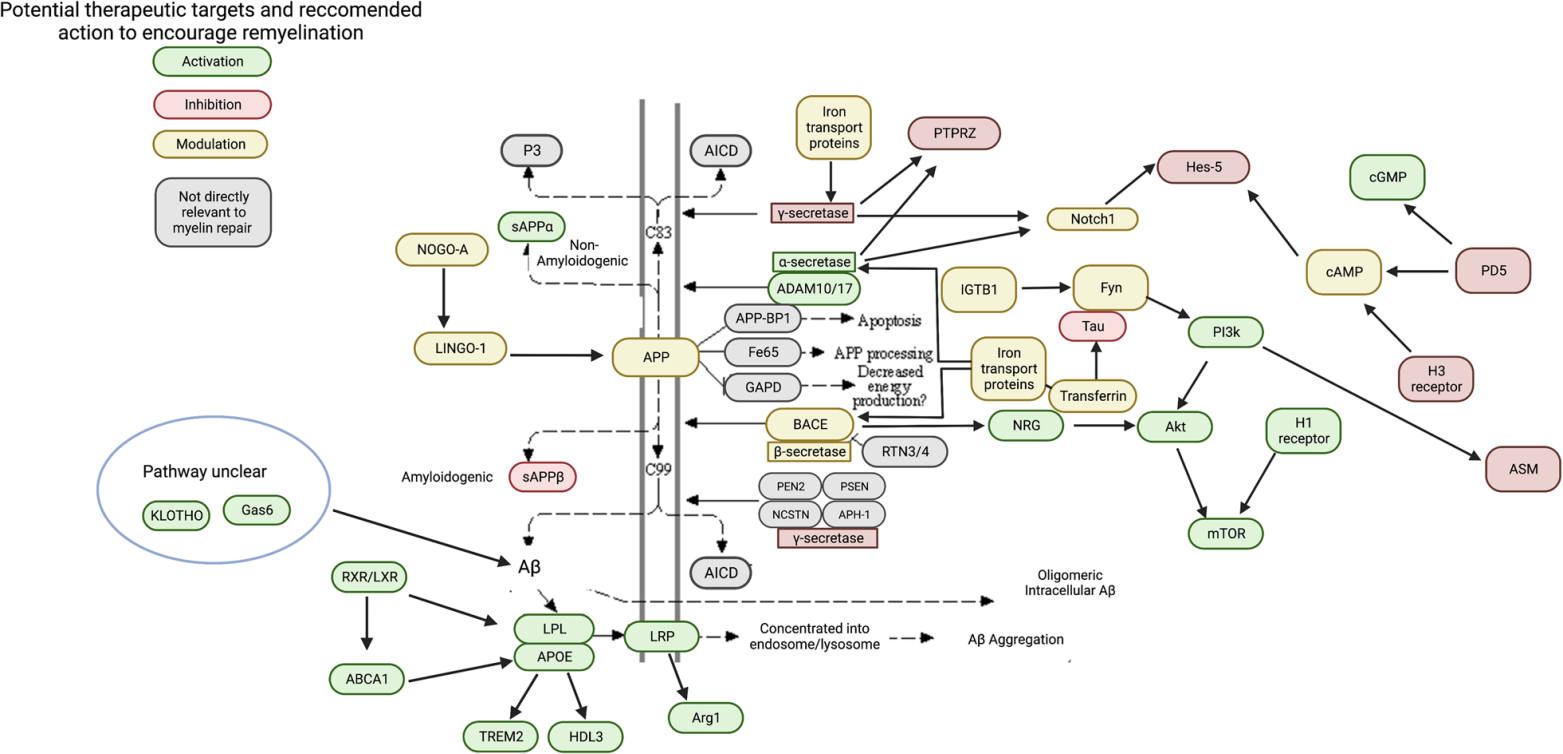
A schematic expanding the canonical KEGG pathway for AD, superimposed with proposed specific modulations that may upregulate myelination based on literature search. Targets are color-coded to indicate what action, based on empirical studies covered in this review, may simultaneously (1) encourage at least part of the remyelination cascade and (2) discourage development of AD pathology simultaneously. Green indicates target activation, red indicates inhibition, and yellow indicates conflicting literature on whether activation or inhibition is beneficial. Gray targets are not relevant in the scope of this review

**Fig. 4:**
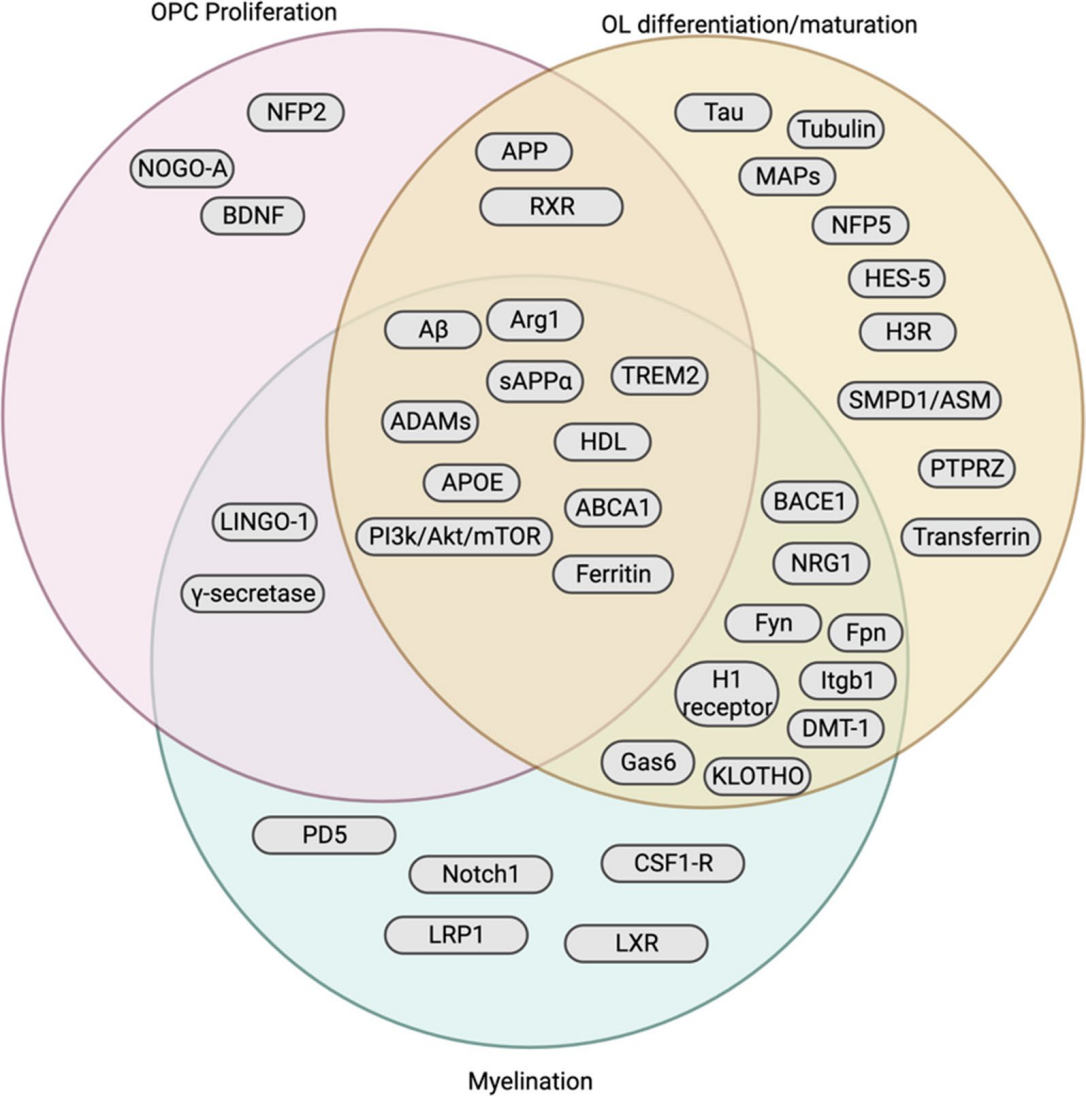
Organization of gene and pathway targets as described within the scope of this review based on their impact on OPC proliferation, OL differentiation/maturation, and/or myelination

**Fig. 5:**
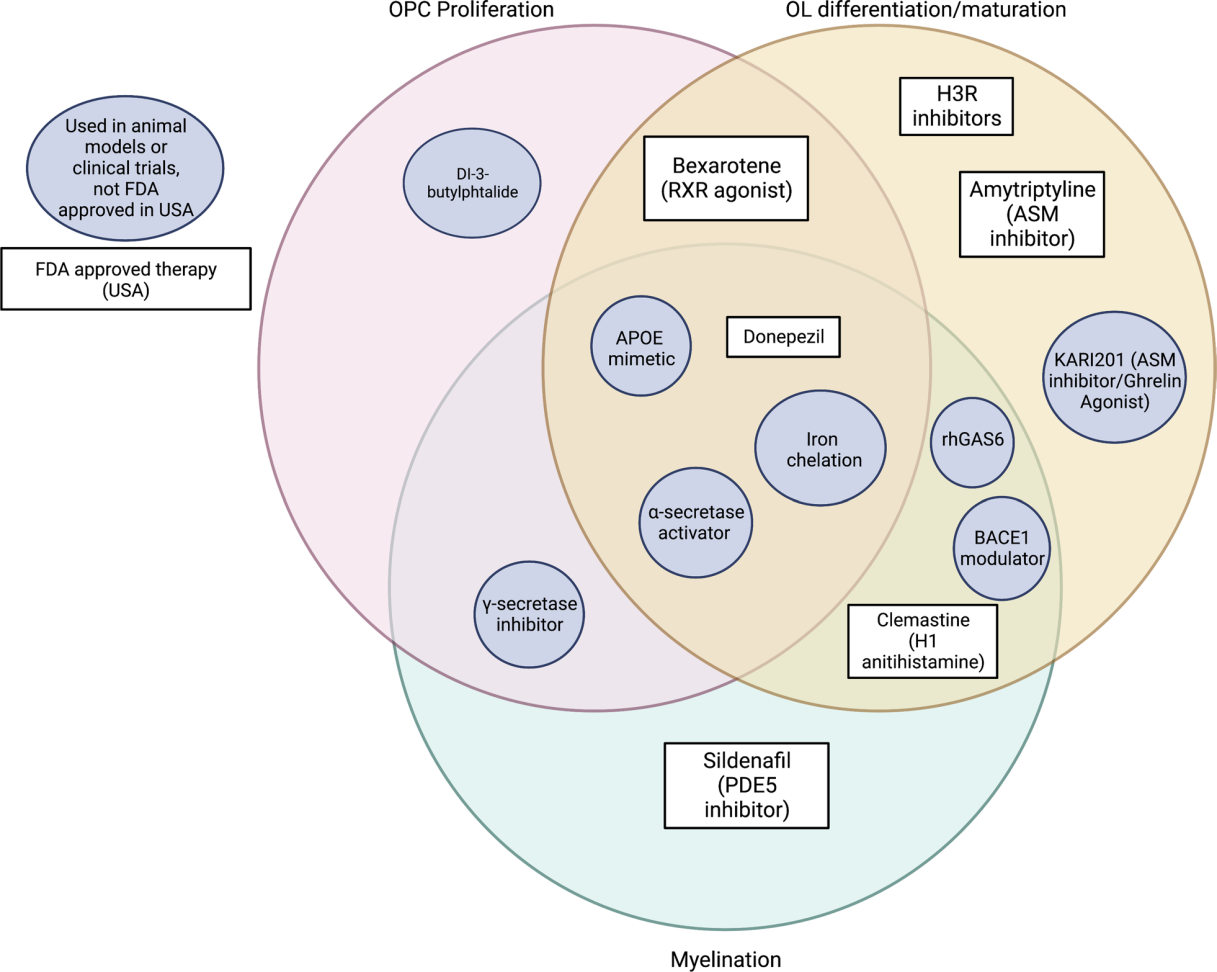
Existing compounds that have been investigated thus far in animal studies and clinical trials (circle) or have FDA-approved formulations (rectangle) by mechanism of impact on OPC proliferation, OL differentiation/maturation, and/or myelination

Pathways directly related to AD, including α-, β-, and γ-secretase pathways, may have dual targets that ameliorate both Aβ and myelin repair mechanisms (Figs. 3, 4 and 5). These pathways may also be synergistically acting with dysfunctional myelin repair mechanisms. Inability of pro-remyelinating α-secretase processing may lead to overwhelming cleavage by β- and γ-secretases, where the excess Aβ may either impair or overpower concomitant β-secretase cleavage of pro-myelinating NRG1. NRG1 inhibition leading to limited remyelination may offer a possible explanation for the failure of BACE1 inhibitors in clinical trials. Moreover, considering the role of NRG1 during BACE1 inhibition may also offer a potential solution, as BACE1 inhibitors may still be used as an anti-amyloid treatment if they are used in tandem with a protein kinase B (Akt) activator.

Additionally, the ABCA1/ApoE/HDL pathway may be critical for remyelination. The effects of ApoE mimetics, which rescue myelin repair while also suppressing macrophagic activity, suggest that macrophages seen in disease-related inflammation may be over-clearing debris. ApoE mimetics can also act to upregulate TREM2, which may increase OPC proliferation and encourage the remyelination cascade. The relationship of ApoE, TREM2, and phagocytic activity to myelin in AD is further emphasized by the presence of a TREM2-dependent WAM. WAM may precede DAM gene signatures, indicating the possibility of upstream myelin pathology in early AD, though more research is needed to confirm this and to elucidate the overlap between the two types [68]. Further research is needed to understand the biological premise for observed ApoE dependence of WAM and if it indeed is related to AD pathophysiology. ApoE mimetics and LXR/RXR agonists may be therapeutically beneficial for targeting these pathways. Further lipidomic analysis is warranted to understand the broader impact of lipid metabolism, such as the implication of glycolipids like sulfatide and ganglioside in demyelination and AD-like cognitive impairment [158–160].

Hyperphosphorylation of tau may be directly related to the inhibition of myelin repair. When p-tau is specifically reduced without affecting the total tau levels, increased myelin repair and improved functional outcomes were observed [84]. Targeting tau, NFP, or the IGTB1/Fyn/Ca2/CAMKII signaling pathway may allow for precise control of the OL lineage and remyelinating properties. The involvement of Fyn with Aβ, tau, and oligodendrocytes implies considerable overlap of AD pathology, myelin repair, and Fyn activity that should be further investigated.

The present review also identifies studies supporting the roles of other pathways and targets in both AD and myelin repair. All targets discussed have been demonstrated to play a role in both AD and remyelination. Further research is warranted into these pathways in the context of AD, as the literature does not unanimously support activation or inhibition of many of the signaling pathways, and studies examining the overlap of myelination and AD pathology are scarce. These targets may offer therapeutic potential alone or in combination with other targeted AD-specific therapeutics, warranting further studies with animal models, single-cell and single-nucleus analysis, and clinical trials when applicable.

This review emphasizes a body of work which demonstrates an overlap between myelin repair mechanisms and AD pathogenesis. However, many studies using preclinical models have fallen short of thoroughly addressing the development of hallmark AD pathology in this shared context. Thus, despite evidence of involvement of myelin repair in different signaling pathways of the amyloid/tau/neurodegeneration cascade, proposing a single, cohesive model is beyond the scope of this review. Limitations regarding scope also include the focus on myelin repair, as it does not comprehensively address mechanisms of injury or maintenance, though other such reviews do cover this topic [9–12]. Much work remains to be done to fully reveal the implications of myelin repair in AD. Due to the urgent need for disease-modifying therapies, it may be possible to reverse-engineer the relationship of myelination with AD progression through therapeutic approaches discussed in this review.

**Fig. 6:**
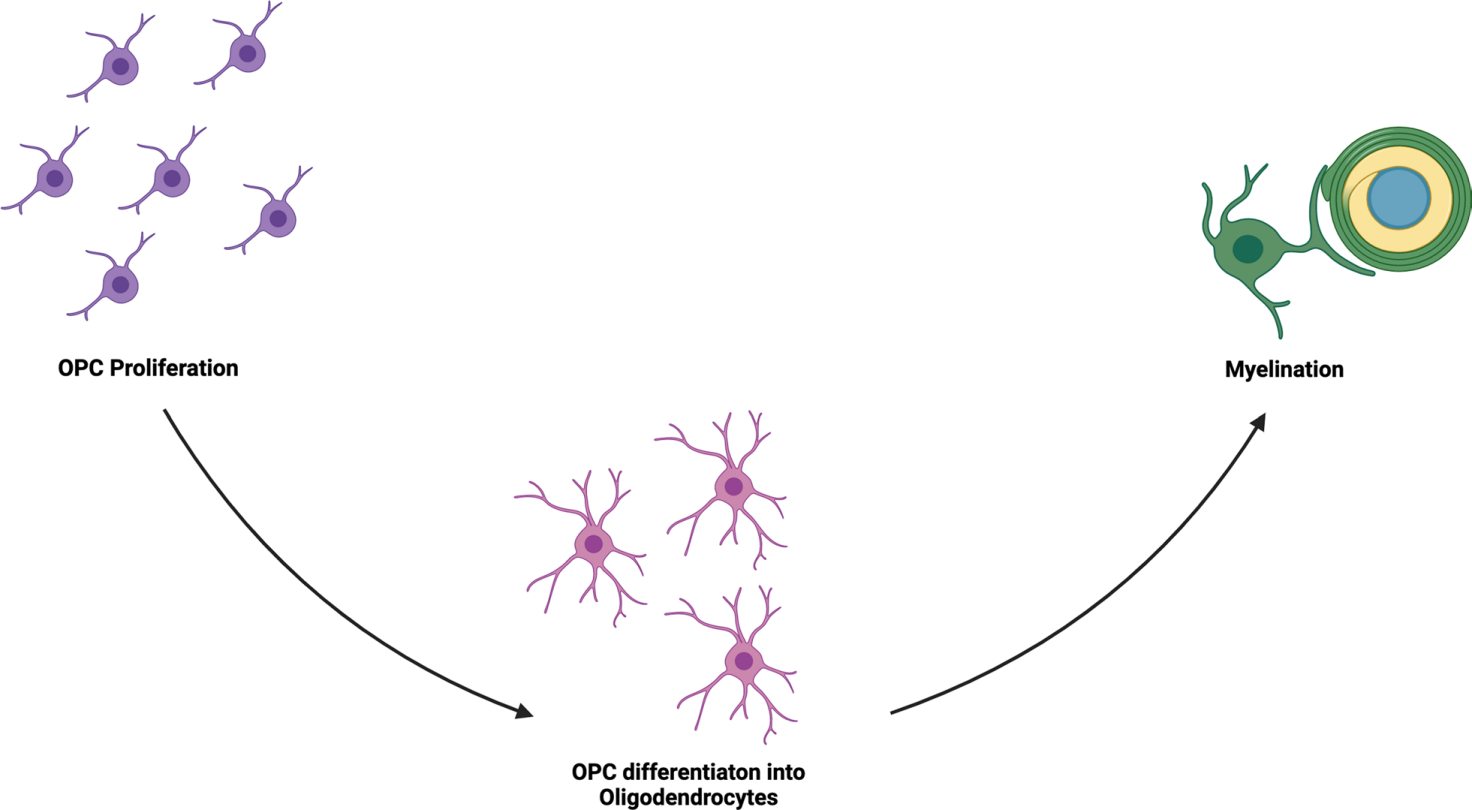
Schematic of the main constituents of myelin repair that can be targeted by pathway modulation: OPC proliferation, OPC differentiation and oligodendrocyte maturation/survival, and the actual formation of myelin sheath and associated proteins

In conclusion, we have shown that there are numerous target pathways that directly overlap with both myelin repair and AD pathophysiology, including APP processing, ApoE signaling, and tau-Fyn processing. Additionally, other pathways and druggable targets have been shown to both ameliorate AD and restore remyelination. From this review, three targets for myelin repair have become apparent: (1) OPC proliferation, (2) OL maturation, and (3) myelin sheath production (Fig. 6). These targets may also be employed to prevent initial myelin damage, although the injurious mechanisms are beyond the scope of this review. More research needs to be done to elucidate which part(s) of these processes are most impaired in AD, if myelin repair dysfunction indeed occurs earlier in the disease course than previously suggested, and which, if any, of the pathways above can ameliorate myelin pathology and potentially restore myelin function.

## Data Availability

Not applicable.

## Abbreviations

ADAM: A disintegrin and metalloproteinase
AChEI: Acetylcholinesterase inhibitor
ASM: Acid sphingomyelinase
AD: Alzheimer’s disease
Aβ: Amyliod beta
APP: Amyloid precursor protein
ApoE: Apolipoprotein E
Arg1: Arginase 1
ABCA1: ATP-binding cassette transporter A1
BACE1: Beta-secretase 1
CREB: CAMP response element-binding protein
DAM: Disease-associated microglia
dl-NBP: Dl-3-butylphtalide
EAE: Experimental autoimmune encephalomyelitis
GAS6: Growth-arrest specific protein 6
HES-5: Hes family BHLH transcription factor 5
HDL: High density lipoprotein
H3R: Histamine 3 receptor
LINGO-1: Leucine rich repeat and Ig domain containing 1
LPL: Lipoprotein lipase
LXR: Liver X receptors
MS: Multiple sclerosis
MBP: Myelin basic protein
NRG1: Neuregulin 1
NFP: Neurofilament protein
OPC: Oligodendrocyte progenitor cells
OL: Oligodendrocytes (s)
PDE5: Phosphodiesterase-5
PI3K: Phosphoinositide 3-kinase
PTPRZ: Protein-tyrosine phosphatase receptor type Z
RXR: Retinoid X receptor
TREM2: Triggering receptor expressed on myeloid cells 2
WAM: White matter associated microglia
